# Supplementary material for: Distinct Replication Kinetics, Cytopathogenicity, and Immune Gene Regulation in Human Microglia Cells Infected with Asian and African Lineages of Zika Virus
Source: Microorganisms. 2024 Sep 5;12(9):1840. doi: 10.3390/microorganisms12091840 (PMC11433722; doi:10.3390/microorganisms12091840)
Supplement: Supplementary file 1 [file microorganisms-12-01840-s001.zip › microorganisms-3178538-supplementary.pdf]

NS1, NS3 and NS5 protein sequence alignment

Figure S1

NS1

```
Consensus            GCSVDFSKKETRCGTGVFVYNDVEAWRDRYKYHPDSPRRLAAVKQAWEDGICGISSVSR   60
AWH65849 - NS1       GCSVDFSKKETRCGTGVFVYNDVEAWRDRYKYHPDSPRRLAAVKQAWEDGICGISSVSR   60
AYI50273 - NS1       GCSVDFSKKETRCGTGVFVYNDVEAWRDRYKYHPDSPRRLAAVKQAWEDGICGISSVSR   60
AYI50274 - NS1       GCSVDFSKKETRCGTGVFVYNDVEAWRDRYKYHPDSPRRLAAVKQAWEDGICGISSVSR   60
AYI50275 - NS1       GCSVDFSKKETRCGTGVFVYNDVEAWRDRYKYHPDSPRRLAAVKQAWEDGICGISSVSR   60
AYI50388 - NS1       GCSVDFSKKETRCGTGVFVYNDVEAWRDRYKYHPDSPRRLAAVKQAWEDGICGISSVSR   60
AMC13911 - NS1       GCSVDFSKKETRCGTGVFVYNDVEAWRDRYKYHPDSPRRLAAVKQAWEDGICGISSVSR   60
AMZ03556 - NS1       GCSVDFSKKETRCGTGVFVYNDVEAWRDRYKYHPDSPRRLAAVKQAWEDGICGISSVSR   60
ANW07476 - NS1       GCSVDFSKKETRCGTGVFVYNDVEAWRDRYKYHPDSPRRLAAVKQAWEDGICGISSVSR   60
QCG74166 - NS1       GCSVDFSKKETRCGTGVFVYNDVEAWRDRYKYHPDSPRRLAAVKQAWEDGICGISSVSR   60
YP_002790881 - NS1   GCSVDFSKKETRCGTGVFIYNDVEAWRDRYKYHPDSPRRLAAVKQAWEEGICGISSVSR   60
AEN75265 - NS1       GCSVDFSKKETRCGTGVFIYNDVEAWRDRYRYHPDSPRRLAAVKQAWEEGICGISSVSR   60
AMR68906 - NS1       GCSVDFSKKETRCGTGVFIYNDVEAWRDRYRYHPDSPRRLAAVKQAWEEGICGISSVSR   60

Consensus            MENIMWRSVEGELNAILEENGVQLTVVVGSVKNPMWRGPQRLPVPVNELPHGWKAWGKSY  120
AWH65849 - NS1       MENIMWRSVEGELNAILEENGVQLTVVVGSVKNPMGRGPQRLPVPVNELPHGWKAWGKSY  120
AYI50273 - NS1       MENIMWRSVEGELNAILEENGVQLTVVVGSVKNPMWRGPQRLPVPVNELPHGWKAWGKSY  120
AYI50274 - NS1       MENIMWRSVEGELNAILEENGVQLTVVVGSVKNPMWRGPQRLPVPVNELPHGWKAWGKSY  120
AYI50275 - NS1       MENIMWRSVEGELNAILEENGVQLTVVVGSVKNPMWRGPQRLPVPVNELPHGWKAWGKSY  120
AYI50388 - NS1       MENIMWRSVEGELNAILEENGVQLTVVVGSVKNPMWRGPQRLPVPVNELPHGWKAWGKSY  120
AMC13911 - NS1       MENIMWRSVEGELNAILEENGVQLTVVVGSVKNPMWRGPQRLPVPVNELPHGWKAWGKSY  120
AMZ03556 - NS1       MENIMWRSVEGELNAILEENGVQLTVVVGSVKNPMWRGPQRLPVPVNELPHGWKAWGKSY  120
ANW07476 - NS1       MENIMWRSVEGELNAILEENGVQLTVVVGSVKNPMWRGPQRLPVPVNELPHGWKAWGKSY  120
QCG74166 - NS1       MENIMWRSVEGELNAILEENGVQLTVVVGSVKNPMWRGPQRLPVPVNELPHGWKAWGKSY  120
YP_002790881 - NS1   MENIMWKSVEGELNAILEENGVQLTVVVGSVKNPMWRGPQRLPVPVNELPHGWKAWGKSY  120
AEN75265 - NS1       MENIMWKSVEGELNAILEENGVQLTVVVGSVKNPMWRGPRRLPVPVNELPHGWKAWGKSY  120
AMR68906 - NS1       MENIMWKSVEGELNAILEENGVQLTVVVGSVKNPMWRGPRRLPVPVNELPHGWKAWGKSY  120

Consensus            FVRAAKTNNSFVVDGDTLKECPLKHRAWNSFLVEDHGFGVFHTSVWLKVREDYSLECDPA  180
AWH65849 - NS1       FVRAAKTNNSFVVDGDTLKECPLKHRAWNSFLVEDHGFGVFHTSVWLKVREDYSLECDPA  180
AYI50273 - NS1       FVRAAKTNNSFVVDGDTLKECPLKHRAWNSFLVEDHGFGVFHTSVWLKVREEYSLECDPA  180
AYI50274 - NS1       FVRAAKTNNSFVVDGDTLKECPLKHRAWNSFLVEDHGFGVFHTSVWLKVREDYSLECDPA  180
AYI50275 - NS1       FVRAAKTNNSFVVDGDTLKECPLKHRAWNSFLVEDHGFGVFHTSVWLKVREDYSLECDPA  180
AYI50388 - NS1       FVRAAKTNNSFVVDGDTLKECPLKHRAWNSFLVEDHGFGVFHTSVWLKVREDYSLECDPA  180
AMC13911 - NS1       FVRAAKTNNSFVVDGDTLKECPLKHRAWNSFLVEDHGFGVFHTSVWLKVREDYSLECDPA  180
AMZ03556 - NS1       FVRAAKTNNSFVVDGDTLKECPLKHRAWNSFLVEDHGFGVFHTSVWLKVREDYSLECDPA  180
ANW07476 - NS1       FVRAAKTNNSFVVDGDTLKECPLKHRAWNSFLVEDHGFGVFHTSVWLKVREDYSLECDPA  180
QCG74166 - NS1       FVRAAKTNNSFVVDGDTLKECPLKHRAWNSFLVEDHGFGVFHTSVWLKVREDYSLECDPA  180
YP_002790881 - NS1   FVRAAKTNNSFVVDGDTLKECPLEHRAWNSFLVEDHGFGVFHTSVWLKVREDYSLECDPA  180
AEN75265 - NS1       FVRAAKTNNSFVVDGDTLKECPLKHRAWNSFLVEDHGFGVFHTSVWLKVREDYSLECDPA  180
AMR68906 - NS1       FVRAAKTNNSFVVDGDTLKECPLKHRAWNSFLVEDHGFGVFHTSVWLKVREDYSLECDPA  180

Consensus            VIGTAVKGKEAVHSDLGYWIESEKNDTWRLKRAHLIEMKTCEWPKSHTLWTDGIEESDLI  240
AWH65849 - NS1       VIGTAVKGKEAVHSDLGYWIESEKNDTWRLKRAHLIEMKTCEWPKSHTLWTDGIEESDLI  240
AYI50273 - NS1       VIGTAVKGKEAVHSDLGYWIESEKNDTWRLKRAHLIEMKTCEWPKSHTLWTDGIEESDLI  240
AYI50274 - NS1       VIGTAVKGKEAVHSDLGYWIESEKNDTWRLKRAHLIEMKTCEWPKSHTLWTDGIEESDLI  240
AYI50275 - NS1       VIGTAVKGKEAVHSDLGYWIESEKNDTWRLKRAHLIETKTCEWPKSHTLWTDGIEESDLI  240
AYI50388 - NS1       VIGTAVKGKEAVHSDLGYWIESEKNDTWRLKRAHLIEMKTCEWPKSHTLWTDGIEESDLI  240
AMC13911 - NS1       VIGTAVKGKEAVHSDLGYWIESEKNDTWRLKRAHLIEMKTCEWPKSHTLWTDGIEESDLI  240
AMZ03556 - NS1       VIGTAVKGKEAVHSDLGYWIESEKNDTWRLKRAHLIEMKTCEWPKSHTLWTDGIEESDLI  240
ANW07476 - NS1       VIGTAVKGKEAVHSDLGYWIESEKNDTWRLKRAHLIEMKTCEWPKSHTLWTDGIEESDLI  240
QCG74166 - NS1       VIGTAVKGKEAVHSDLGYWIESEKNDTWRLKRAHLIEMKTCEWPKSHTLWTDGIEESDLI  240
YP_002790881 - NS1   VIGTAVKGREAAHSDLGYWIESEKNDTWRLKRAHLIEMKTCEWPKSHTLWTDGVEESDLI  240
AEN75265 - NS1       VIGTAVKGKEAAHSDLGYWIESEKNDTWRLKRAHLIEMKTCEWPKSHTLWTDGVEESDLI  240
AMR68906 - NS1       VIGTAVKGKEAAHSDLGYWIESEKNDTWRLKRAHLIEMKTCEWPKSHTLWTDGVEESDLI  240

Consensus            IPKSLAGPLSHHNTREGYRTQMKGPWHSEELEIRFEECPGTKVHVEETCGTRGPSLRSTT  300
```

```
AWH65849 - NS1     IPKSLAGPLSHHNTREGYRTQMKGPWHSEELEIRFEECPGTKVHVEETCGTRGPSLRSTT  300
AYI50273 - NS1     IPKSLAGPLSHHNTREGYRTQMKGPWHSEELEIRFEECPGTKVHVEETCGTRGPSLRSTT  300
AYI50274 - NS1     IPTSLAGPLSHHNTREGYRTQMKGPWHSEELEIRFEECPGTKVHVEETCGTRGPSLRSTT  300
AYI50275 - NS1     IPKSLAGPLSHHNTREGYRTQMKGPWHSEELEIRFEECPGTKVHVEETCGTRGPSLRSTT  300
AYI50388 - NS1     IPKSLAGPLSHHNTREGYRTQMEGPWHSEELEIRFEECPGTKVHVEETCGTRGPSLRSTT  300
AMC13911 - NS1     IPKSLAGPLSHHNTREGYRTQMKGPWHSEELEIRFEECPGTKVHVEETCGTRGPSLRSTT  300
AMZ03556 - NS1     IPKSLAGPLSHHNTREGYRTQMKGPWHSEELEIRFEECPGTKVHVEETCGTRGPSLRSTT  300
ANW07476 - NS1     IPKSLAGPLSHHNTREGYRTQMKGPWHSEELEIRFEECPGTKVHVEETCGTRGPSLRSTT  300
QCG74166 - NS1     IPKSLAGPLSHHNTREGYRTQMKGPWHSEELEIRFEECPGTKVHVEETCGTRGPSLRSTT  300
YP_002790881 - NS1 IPKSLAGPLSHHNTREGYRTQVKGPWHSEELEIRFEECPGTKVYVEETCGTRGPSLRSTT  300
AEN75265 - NS1     IPKSLAGPLSHHNTREGYRTQVKGPWHSEELEIRFEECPGTKVHVEETCGTRGPSLRSTT  300
AMR68906 - NS1     IPKSLAGPLSHHNTREGYRTQVKGPWHSEELEIRFEECPGTKVHVEETCGTRGPSLRSTT  300

Consensus          ASGRVIEEWCCRECTMPPLSFRAKDGCWYGMEIRPRKEPESNLVRSMVTAGS          352
AWH65849 - NS1     ASGRVIEEWCCRECTMPPLSFRAKDGCWYGMEIRPRKEPESNLVRSMVTAGS          352
AYI50273 - NS1     ASGRVIEEWCCRECTMPPLSFRAKDGCWYGMEIRPRKEPESNLVRSMVTAGS          352
AYI50274 - NS1     ASGRVIEEWCCRECTMPPLSFRAKDGCWYGMEIRPRKEPESNLVRSMVTAGS          352
AYI50275 - NS1     ASGRVIEEWCCRECTMPPLSFRAKDGCWYGMEIRPRKEPESNLVRSMVTAGS          352
AYI50388 - NS1     ASGRVIEEWCCRECTMPPLSFRAKDGCWYGMEIRPRKEPESNLVRSMVTAGS          352
AMC13911 - NS1     ASGRVIEEWCCRECTMPPLSFRAKDGCWYGMEIRPRKEPESNLVRSMVTAGS          352
AMZ03556 - NS1     ASGRVIEEWCCRECTMPPLSFRAKDGCWYGMEIRPRKEPESNLVRSMVTAGS          352
ANW07476 - NS1     ASGRVIEEWCCRECTMPPLSFRAKDGCWYGMEIRPRKEPESNLVRSMVTAGS          352
QCG74166 - NS1     ASGRVIEEWCCRECTMPPLSFRAKDGCWYGMEIRPRKEPESNLVRSMVTAGS          352
YP_002790881 - NS1 ASGRVIEEWCCRECTMPPLSFRAKDGCWYGMEIRPRKEPESNLVRSMVTAGS          352
AEN75265 - NS1     ASGRVIEEWCCRECTMPPLSFRAKDGCWYGMEIRPRKEPESNLVRSMVTAGS          352
AMR68906 - NS1     ASGRVIEEWCCRECTMPPLSFRAKDGCWYGMEIRPRKEPESNLVRSMVTAGS          352
```

NS2A

```
Consensus           GVLVILLMVQEGLKKRMTTKIIISTSMAVLVAMILGGFSMSDLAKLAILMGATFAEMNTG  60
AYI50388 - NS2A     GVLVILLMVQEGLKKRMTTKIIISTSMAVLVAMILGGFSMSDLAKLAILMGATFAEMNTG  60
AYI50275 - NS2A     GVLVILLMVQEGLKKRMTTKIIISTSMAVLVAMILGGFSMSDLAKLAILMGATFAEMNTG  60
AWH65849 - NS2A     GVLVILLMVQEGLKKRMTTKIIISTSMAVLVAMILGGFSMSDLAKLAILMGATFAEMNTG  60
AMC13911 - NS2A     GVLVILLMVQEGLKKRMTTKIIISTSMAVLVAMILGGFSMSDLAKLAILMGATFAEMNTG  60
AYI50274 - NS2A     GVLVILLMVQEGLKKRMTTKIIISTSMAVLVAMILGGFSMSDLAKLAILMGATFAEMNTG  60
QCG74166 - NS2A     GVLVILLMVQEGLKKRMTTKIIISTSMAVLVAMILGGFSMSDLAKLAILMGATFAEMNTG  60
ANW07476 - NS2A     GVLVILLMVQEGLKKRMTTKIIISTSMAVLVAMILGGFSMSDLAKLAILMGATFAEMNTG  60
AMZ03556 - NS2A     GVLVILLMVQEGLKKRMTTKIIISTSMAVLVAMILGGFSMSDLAKLAILMGATFAEMNTG  60
AYI50273 - NS2A     GVLVILLMVQEGLKKRMTTKIIISTSMAVLVAMILGGFSMSDLAKLAILMGATFAEMNTG  60
YP_002790881 - NS2A GVLVILLMVQEGLKKRMTTKIIMSTSMAVLVVMILGGFSMSDLAKLVILMGATFAEMNTG  60
AEN75265 - NS2A     GVLVILLMVQEGLKKRMTTKIIMSTSMAMLVAMVLGGFSMSDLAKLVILMGATFAEMNTG  60
AMR68906 - NS2A     GVLVILLMVQEGLKKRMTTKIIMSTSMAMLVAMVLGGFSMSDLAKLVILMGATFAEMNTG  60

Consensus           GDVAHLALIAAFKVRPALLVSFIFRANWTPRESMLLALASCLLQTAISALEGDLMVLING  120
AYI50388 - NS2A     GDVAHLALIAAFKVRPALLVSFIFRANWTPRESMLLALASCLLQTAISALEGDLMVLING  120
AYI50275 - NS2A     GDVAHLALIAAFKVRPALLVSFIFRANWTPRESMLLALASCLLQTAISALEGDLMVLING  120
AWH65849 - NS2A     GDVAHLALIAAFKVRPALLVSFIFRANWTPRESMLLALASCLLQTAISALEGDLMVLING  120
AMC13911 - NS2A     GDVAHLALIAAFKVRPALLVSFIFRANWTPRESMLLALASCLLQTAISALEGDLMVLING  120
AYI50274 - NS2A     GDVAHLALIAAFKVRPALLVSFIFRANWTPRESMLLALASCLLQTAISALEGDLMVLING  120
QCG74166 - NS2A     GDVAHLALIAAFKVRPALLVSFIFRANWTPRESMLLALASCLLQTAISALEGDLMVLING  120
ANW07476 - NS2A     GDVAHLALIAAFKVRPALLVSFIFRANWTPRESMLLALASCLLQTAISALEGDLMVLING  120
AMZ03556 - NS2A     GDVAHLALIAAFKVRPALLVSFIFRANWTPRESMLLALASCLLQTAISALEGDLMVLING  120
AYI50273 - NS2A     GDVAHLALIAAFKVRPALLVSFIFRANWTPRESMLLALASCLLQTAISALEGDLMVLING  120
YP_002790881 - NS2A GDVAHLALVAAFKVRPALLVSFIFRANWTPRESMLLALASCLLQTAISALEGDLMVLING  120
AEN75265 - NS2A     GDVAHLALVAAFKVRPALLVSFIFRANWTPRESMLLALASCLLQTAISALEGELMVLVNG  120
AMR68906 - NS2A     GDVAHLALVAAFKVRPALLVSFIFRANWTPRESMLLALASCLLQTAISALEGELMVLVNG  120

Consensus           FALAWLAIRAMVVPRTDNITLAILAALTPLARGTLLVAWRAGLATCGGFMLLSLKGKGSV  180
AYI50388 - NS2A     FALAWLAIRAMVVPRTDNITLAILAALTPLARGTLLVAWRAGLATCGGFMLLSLKGKGSV  180
AYI50275 - NS2A     FALAWLAIRAMVVPRTDNITLAILAALTPLARGTLLVAWRAGLATCGGFMLLSLKGKGSV  180
AWH65849 - NS2A     FALAWLAIRAMVVPRTDNITLAILAALTPLARGTLLVAWRAGLATCGGFMLLSLKGKGSV  180
AMC13911 - NS2A     FALAWLAIRAMVVPRTDNITLAILAALTPLARGTLLVAWRAGLATCGGFMLLSLKGKGSV  180
AYI50274 - NS2A     FALAWLAIRAMVVPRTDNITLAILAALTPLARGTLLVAWRAGLATCGGFMLLSLKGKGSV  180
```

```
QCG74166 - NS2A     FALAWLAIRAMVVPRTDNITLAILAALTPLARGTLLVAWRAGLATCGGFMLLSLKGKGSV  180
ANW07476 - NS2A     FALAWLAIRAMVVPRTDNITLAILAALTPLARGTLLVAWRAGLATCGGFMLLSLKGKGSV  180
AMZ03556 - NS2A     FALAWLAIRAMVVPRTDNITLAILAALTPLARGTLLVAWRAGLATCGGFMLLSLKGKGSV  180
AYI50273 - NS2A     FALAWLAIRAMVVPRTDNITLAILAALTPLARGTLLVAWRAGLATCGGFMLLSLKGKGSV  180
YP_002790881 - NS2A FALAWLAIRAMAVPRTDNIALPILAALTPLARGTLLVAWRAGLATCGGIMLLSLKGKGSV  180
AEN75265 - NS2A     FALAWLAIRAMAVPRTDNIALAILAALTPLARGTLLVAWRAGLATCGGFMLISLKGKGSV  180
AMR68906 - NS2A     FALAWLAIRAMAVPRTDNIALAILAALTPLARGTLLVAWRAGLATCGGFMLISLKGKGSV  180

Consensus           KKNLPFVMALGLTAVRLVDPINVVGLLLLTRSGKR                           215
AYI50388 - NS2A     KKNLPFVMALGLTAVRLVDPINVVGLLLLTRSGKR                           215
AYI50275 - NS2A     KKNLPFVMALGLTAVRLVDPINVVGLLLLTRSGKR                           215
AWH65849 - NS2A     KKNLPFVMALGLTAVRLVDPINVVGLLLLTRSGKR                           215
AMC13911 - NS2A     KKNLPFVMALGLTAVRLVDPINVVGLLLLTRSGKR                           215
AYI50274 - NS2A     KKNLPFVMALGLTAVRLVDPINVVGLLLLTRSGKR                           215
QCG74166 - NS2A     KKNLPFVMALGLTAVRLVDPINVVGLLLLTRSGKR                           215
ANW07476 - NS2A     KKNLPFVMALGLTAVRLVDPINVVGLLLLTRSGKR                           215
AMZ03556 - NS2A     KKNLPFVMALGLTAVRLVDPINVVGLLLLTRSGKR                           215
AYI50273 - NS2A     KKNLPFVMALGLTAVRLVDPINVVGLLLLTRSGKR                           215
YP_002790881 - NS2A KKNLPFVMALGLTAVRVVDPINVVGLLLLTRSGKR                           215
AEN75265 - NS2A     KKNLPFVMALGLTAVRIVDPINVVGLLLLTRSGKR                           215
AMR68906 - NS2A     KKNLPFVMALGLTAVRIVDPINVVGLLLLTRSGKR                           215
```

NS3

```
Consensus          SGALWDVPAPKEVKKGETTDGVYRVMTRRLLGSTQVGVGVMQEGVFHTMWHVTKGSALRS  60
AMC13911 - NS3     SGALWDVPAPKEVKKGETTDGVYRVMTRRLLGSTQVGVGVMQEGVFHTMWHVTKGSALRS  60
AMZ03556 - NS3     SGALWDVPAPKEVKKGETTDGVYRVMTRRLLGSTQVGVGVMQEGVFHTMWHVTKGSALRS  60
ANW07476 - NS3     SGALWDVPAPKEVKKGETTDGVYRVMTRRLLGSTQVGVGVMQEGVFHTMWHVTKGSALRS  60
AWH65849 - NS3     SGALWDVPAPKEVKKGETTDGVYRVMTRRLLGSTQVGVGVMQEGVFHTMWHVTKGSALRS  60
AYI50273 - NS3     SGALWDVPAPKEVKKGETTDGVYRVMTRRLLGSTQVGVGVMQEGVFHTMWHVTKGSALRS  60
AYI50274 - NS3     SGALWDVPAPKEVKKGETTDGVYRVMTRRLLGSTQVGVGVMQEGVFHTMWHVTKGSALRS  60
AYI50275 - NS3     SGALWDVPAPKEVKKGETTDGVYRVMTRRLLGSTQVGVGVMQEGVFHTMWHVTKGSALRS  60
AYI50388 - NS3     SGALWDVPAPKEVKKGETTDGVYRVMTRRLLGSTQVGVGVMQEGVFHTMWHVTKGSALRS  60
QCG74166 - NS3     SGALWDVPAPKEVKKGETTDGVYRVMTRRLLGSTQVGVGVMQEGVFHTMWHVTKGSALRS  60
YP_002790881 - NS3 SGALWDVPAPKEVKKGETTDGVYRVMTRRLLGSTQVGVGVMQEGVFHTMWHVTKGAALRS  60
AEN75265 - NS3     SGALWDVPAPKEVKKGETTDGVYRVMTRRLLGSTQVGVGVMQEGVFHTMWHVTKGAALRS  60
AMR68906 - NS3     SGALWDVPAPKEVKKGETTDGVYRVMTRRLLGSTQVGVGVMQEGVFHTMWHVTKGAALRS  60

Consensus          GEGRLDPYWGDVKQDLVSYCGPWKLDAAWDGHSEVQLLAVPPGERARNIQTLPGIFKTKD  120
AMC13911 - NS3     GEGRLDPYWGDVKQDLVSYCGPWKLDAAWDGHSEVQLLAVPPGERARNIQTLPGIFKTKD  120
AMZ03556 - NS3     GEGRLDPYWGDVKQDLVSYCGPWKLDAAWDGHSEVQLLAVPPGERARNIQTLPGIFKTKD  120
ANW07476 - NS3     GEGRLDPYWGDVKQDLVSYCGPWKLDAAWDGHSEVQLLAVPPGERARNIQTLPGIFKTKD  120
AWH65849 - NS3     GEGRLDPYWGDVKQDLVSYCGPWKLDAAWDGHSEVQLLAVPPGERARNIQTLPGIFKTKD  120
AYI50273 - NS3     GEGRLDPYWGDVKQDLVSYCGPWKLDAAWDGHSEVQLLAVPPGERARNIQTLPGIFKTKD  120
AYI50274 - NS3     GEGRLDPYWGDVKQDLVSYCGPWKLDAAWDGHSEVQLLAVPPGERARNIQTLPGIFKTKD  120
AYI50275 - NS3     GEGRLDPYWGDVKQDLVSYCGPWKLDAAWDGHSEVQLLAVPPGERARNIQTLPGIFKTKD  120
AYI50388 - NS3     GEGRLDPYWGDVKQDLVSYCGPWKLDAAWDGHSEVQLLAVPPGERARNIQTLPGIFKTKD  120
QCG74166 - NS3     GEGRLDPYWGDVKQDLVSYCGPWKLDAAWDGHSEVQLLAVPPGERARNIQTLPGIFKTKD  120
YP_002790881 - NS3 GEGRLDPYWGDVKQDLVSYCGPWKLDAAWDGLSEVQLLAVPPGERARNIQTLPGIFKTKD  120
AEN75265 - NS3     GEGRLDPYWGDVKQDLVSYCGPWKLDAAWDGLSEVQLLAVPPGERAKNIQTLPGIFKTKD  120
AMR68906 - NS3     GEGRLDPYWGDVKQDLVSYCGPWKLDAAWDGLSEVQLLAVPPGERAKNIQTLPGIFKTKD  120

Consensus          GDIGAVALDYPAGTSGSPILDKCGRVIGLYGNGVVIKNGSYVSAITQGRREEETPVECFE  180
AMC13911 - NS3     GDIGAVALDYPAGTSGSPILDKCGRVIGLYGNGVVIKNGSYVSAITQGRREEETPVECFE  180
AMZ03556 - NS3     GDIGAVALDYPAGTSGSPILDKCGRVIGLYGNGVVIKNGSYVSAITQGRREEETPVECFE  180
ANW07476 - NS3     GDIGAVALDYPAGTSGSPILDKCGRVIGLYGNGVVIKNGSYVSAITQGRREEETPVECFE  180
AWH65849 - NS3     GDIGAVALDYPAGTSGSPILDKCGRVIGLYGNGVVIKNGSYVSAITQGRREEETPVECFE  180
AYI50273 - NS3     GDIGAVALDYPAGTSGSPILDKCGRVIGLYGNGVVIKNGSYVSAITQGRREEETPVECFE  180
AYI50274 - NS3     GDIGAVALDYPAGTSGSPILDKCGRVIGLYGNGVVIKNGSYVSAITQGRREEETPVECFE  180
AYI50275 - NS3     GDIGAVALDYPAGTSGSPILDKCGRVIGLYGNGVVIKNGSYVSAITQGRREEETPVECFE  180
AYI50388 - NS3     GDIGAVALDYPAGTSGSPILDKCGRVIGLYGNGVVIKNGSYVSAITQGRREEETPVECFE  180
QCG74166 - NS3     GDIGAVALDYPAGTSGSPILDKCGRVIGLYGNGVVIKNGSYVSAITQGRREEETPVECFE  180
YP_002790881 - NS3 GDIGAVALDYPAGTSGSPILDKCGRVIGLYGNGVVIKNGSYVSAITQGKREEETPVECFE  180
```

```
AEN75265 - NS3     GDIGAVALDYPAGTSGSPILDKCGRVIGLYGNGVVIKNGSYVSAITQGKREEETPVECFE  180
AMR68906 - NS3     GDIGAVALDYPAGTSGSPILDKCGRVIGLYGNGVVIKNGSYVSAITQGKREEETPVECFE  180

Consensus          PSMLKKKQLTVLDLHPGAGKTRRVLPEIVREAIKTRLRTVILAPTRVVAEMEEALRGLP  240
AMC13911 - NS3     PSMLKKKQLTVLDLHPGAGKTRRVLPEIVREAIKTRLRTVILAPTRVVAEMEEALRGLP  240
AMZ03556 - NS3     PSMLKKKQLTVLDLHPGAGKTRRVLPEIVREAIKTRLRTVILAPTRVVAEMEEALRGLP  240
ANW07476 - NS3     PSMLKKKQLTVLDLHPGAGKTRRVLPEIVREAIKTRLRTVILAPTRVVAEMEEALRGLP  240
AWH65849 - NS3     PSMLKKKQLTVLDLHPGAGKTRRVLPEIVREAIKTRLRTVILAPTRVVAEMEEALRGLP  240
AYI50273 - NS3     PSMLKKKQLTVLDLHPGAGKTRRVLPEIVREAIKTRLRTVILAPTRVVAEMEEALRGLP  240
AYI50274 - NS3     PSMLKKKQLTVLDLHPGAGKTRRVLPEIVREAIKTRLRTVILAPTRVVAEMEEALRGLP  240
AYI50275 - NS3     PSMLKKKQLTVLDLHPGAGKTRRVLPEIVREAIKTRLRTVILAPTRVVAEMEEALRGLP  240
AYI50388 - NS3     PSMLKKKQLTVLDLHPGAGKTRRVLPEIVREAIKTRLRTVILAPTRVVAEMEEALRGLP  240
QCG74166 - NS3     PSMLKKKQLTVLDLHPGAGKTRRVLPEIVREAIKTRLRTVILAPTRVVAEMEEALRGLP  240
YP_002790881 - NS3 PSMLKKKQLTVLDLHPGAGKTRRVLPEIVREAIKKRLRTVILAPTRVVAEMEEALRGLP  240
AEN75265 - NS3     PSMLRKKQLTVLDLHPGAGKTRRVLPEIVREAIKKRLRTVILAPTRVVAEMEEALRGLP  240
AMR68906 - NS3     PSMLRKKQLTVLDLHPGAGKTRRVLPEIVREAIKKRLRTVILAPTRVVAEMEEALRGLP  240

Consensus          VRYMTTAVNVTHSGTEIVDLMCHATFTSRLLQPIRVPNYNLYIMDEAHFTDPSSIAARGY  300
AMC13911 - NS3     VRYMTTAVNVTHSGTEIVDLMCHATFTSRLLQPIRVPNYNLYIMDEAHFTDPSSIAARGY  300
AMZ03556 - NS3     VRYMTTAVNVTHSGTEIVDLMCHATFTSRLLQPIRVPNYNLYIMDEAHFTDPSSIAARGY  300
ANW07476 - NS3     VRYMTTAVNVTHSGTEIVDLMCHATFTSRLLQPIRVPNYNLYIMDEAHFTDPSSIAARGY  300
AWH65849 - NS3     VRYMTTAVNVTHSGTEIVDLMCHATFTSRLLQPIRVPNYNLYIMDEAHFTDPSSIAARGY  300
AYI50273 - NS3     VRYMTTAVNVTHSGTEIVDLMCHATFTSRLLQPIRVPNYNLYIMDEAHFTDPSSIAARGY  300
AYI50274 - NS3     VRYMTTAVNVTHSGTEIVDLMCHATFTSRLLQPIRVPNYNLYIMDEAHFTDPSSIAARGY  300
AYI50275 - NS3     VRYMTTAVNVTHSGTEIVDLMCHATFTSRLLQPIRVPNYNLYIMDEAHFTDPSSIAARGY  300
AYI50388 - NS3     VRYMTTAVNVTHSGTEIVDLMCHATFTSRLLQPIRVPNYNLYIMDEAHFTDPSSIAARGY  300
QCG74166 - NS3     VRYMTTAVNVTHSGTEIVDLMCHATFTSRLLQPIRVPNYNLYIMDEAHFTDPSSIAARGY  300
YP_002790881 - NS3 VRYMTTAVNVTHSGTEIVDLMCHATFTSRLLQPIRVPNYNLNIMDEAHFTDPSSIAARGY  300
AEN75265 - NS3     VRYMTTAVNVTHSGTEIVDLMCHATFTSRLLQPIRVPNYNLYIMDEAHFTDPSSIAARGY  300
AMR68906 - NS3     VRYMTTAVNVTHSGTEIVDLMCHATFTSRLLQPIRVPNYNLYIMDEAHFTDPSSIAARGY  300

Consensus          ISTRVEMGEAAAIFMTATPPGTRDAFPDSNSPIMDTEVEVPERAWSSGFDWVTDHSGKTV  360
AMC13911 - NS3     ISTRVEMGEAAAIFMTATPPGTRDAFPDSNSPIMDTEVEVPERAWSSGFDWVTDHSGKTV  360
AMZ03556 - NS3     ISTRVEMGEAAAIFMTATPPGTRDAFPDSNSPIMDTEVEVPERAWSSGFDWVTDHSGKTV  360
ANW07476 - NS3     ISTRVEMGEAAAIFMTATPPGTRDAFPDSNSPIMDTEVEVPERAWSSGFDWVTDHSGKTV  360
AWH65849 - NS3     ISTRVEMGEAAAIFMTATPPGTRDAFPDSNSPIMDTEVEVPERAWSSGFDWVTDHSGKTV  360
AYI50273 - NS3     ISTRVEMGEAAAIFMTATPPGTRDAFPDSNSPIMDTEVEVPERAWSSGFDWVTDHSGKTV  360
AYI50274 - NS3     ISTRVEMGEAAAIFMTATPPGTRDAFPDSNSPIMDTEVEVPERAWSSGFDWVTDHSGKTV  360
AYI50275 - NS3     ISTRVEMGEAAAIFMTATPPGTRDAFPDSNSPIMDTEVEVPERAWSSGFDWVTDHSGKTV  360
AYI50388 - NS3     ISTRVEMGEAAAIFMTATPPGTRDAFPDSNSPIMDTEVEVPERAWSSGFDWVTDHSGKTV  360
QCG74166 - NS3     ISTRVEMGEAAAIFMTATPPGTRDAFPDSNSPIMDTEVEVPERAWSSGFDWVTDHSGKTV  360
YP_002790881 - NS3 ISTRVEMGEAAAIFMTATPPGTRDAFPDSNSPIMDTEVEVPERAWSSGFDWVTDHSGKTV  360
AEN75265 - NS3     ISTRVEMGEAAAIFMTATPPGTRDAFPDSNSPIMDTEVEVPERAWSSGFDWVTDHSGKTI  360
AMR68906 - NS3     ISTRVEMGEAAAIFMTATPPGTRDAFPDSNSPIMDTEVEVPERAWSSGFDWVTDHSGKTI  360

Consensus          WFVPSVRNGNEIAACLTKAGKRVIQLSRKTFETEFQKTKHQEWDFVVTTDISEMGANFKA  420
AMC13911 - NS3     WFVPSVRNGNEIAACLTKAGKRVIQLSRKTFETEFQKTKHQEWDFVVTTDISEMGANFKA  420
AMZ03556 - NS3     WFVPSVRNGNEIAACLTKAGKRVIQLSRKTFETEFQKTKHQEWDFVVTTDISEMGANFKA  420
ANW07476 - NS3     WFVPSVRNGNEIAACLTKAGKRVIQLSRKTFETEFQKTKHQEWDFVVTTDISEMGANFKA  420
AWH65849 - NS3     WFVPSVRNGNEIAACLTKAGKRVIQLSRKTFETEFQKTKHQEWDFVVTTDISEMGANFKA  420
AYI50273 - NS3     WFVPSVRNGNEIAACLTKAGKRVIQLSRKTFETEFQKTKHQEWDFVVTTDISEMGANFKA  420
AYI50274 - NS3     WFVPSVRNGNEIAACLTKAGKRVIQLSRKTFETEFQKTKHQEWDFVVTTDISEMGANFKA  420
AYI50275 - NS3     WFVPSVRNGNEIAACLTKAGKRVIQLSRKTFETEFQKTKHQEWDFVVTTDISEMGANFKA  420
AYI50388 - NS3     WFVPSVRNGNEIAACLTKAGKRVIQLSRKTFETEFQKTKHQEWDFVVTTDISEMGANFKA  420
QCG74166 - NS3     WFVPSVRNGNEIAACLTKAGKRVIQLSRKTFETEFQKTKHQEWDFVVTTDISEMGANFKA  420
YP_002790881 - NS3 WFVPSVRNGNEIAACLTKAGKRVIQLSRKTFETEFQKTKNQEWDFVITTDISEMGANFKA  420
AEN75265 - NS3     WFVPSVRNGNEIAACLTKAGKRVIQLSRKTFETEFQKTKNQEWDFVITTDISEMGANFKA  420
AMR68906 - NS3     WFVPSVRNGNEIAACLTKAGKRVIQLSRKTFETEFQKTKNQEWDFVITTDISEMGANFKA  420

Consensus          DRVIDSRRCLKPVILDGERVILAGPMPVTHASAAQRRGRIGRNPNKPGDEYLYGGGCAET  480
AMC13911 - NS3     DRVIDSRRCLKPVILDGERVILAGPMPVTHASAAQRRGRIGRNPNKPGDEYLYGGGCAET  480
AMZ03556 - NS3     DRVIDSRRCLKPVILDGERVILAGPMPVTHASAAQRRGRIGRNPNKPGDEYLYGGGCAET  480
ANW07476 - NS3     DRVIDSRRCLKPVILDGERVILAGPMPVTHASAAQRRGRIGRNPNKPGDEYLYGGGCAET  480
```

```
AWH65849 - NS3      DRVIDSRRCLKPVILDGERVILAGPMPVTHASAAQRRGRIGRNPNKPGDEYLYGGGCAET  480
AYI50273 - NS3      DRVIDSRRCLKPVILDGERVILAGPMPVTHASAAQRRGRIGRNPNKPGDEYLYGGGCAET  480
AYI50274 - NS3      DRVIDSRRCLKPVILDGERVILAGPMPVTHASAAQRRGRIGRNPNKPGDEYLYGGGCAET  480
AYI50275 - NS3      DRVIDSRRCLKPVILDGERVILAGPMPVTHASAAQRRGRIGRNPNKPGDEYLYGGGCAET  480
AYI50388 - NS3      DRVIDSRRCLKPVILDGERVILAGPMPVTHASAAQRRGRIGRNPNKPGDEYLYGGGCAET  480
QCG74166 - NS3      DRVIDSRRCLKPVILDGERVILAGPMPVTHASAAQRRGRIGRNPNKPGDEYLYGGGCAET  480
YP_002790881 - NS3  DRVIDSRRCLKPVILDGERVILAGPMPVTHASAAQRRGRIGRNPNKPGDEYMYGGGCAET  480
AEN75265 - NS3      DRVIDSRRCLKPVILDGERVILAGPMPVTHASAAQRRGRIGRNPNKPGDEYMYGGGCAET  480
AMR68906 - NS3      DRVIDSRRCLKPVILDGERVILAGPMPVTHASAAQRRGRIGRNPNKPGDEYMYGGGCAET  480

Consensus           DEDHAHWLEARMLLDNIYLQDGLIASLYRPEADKVAAIEGEFKLRTEQRKTFVELMKRGD  540
AMC13911 - NS3      DEDHAHWLEARMLLDNIYLQDGLIASLYRPEADKVAAIEGEFKLRTEQRKTFVELMKRGD  540
AMZ03556 - NS3      DEDHAHWLEARMLLDNIYLQDGLIASLYRPEADKVAAIEGEFKLRTEQRKTFVELMKRGD  540
ANW07476 - NS3      DEDHAHWLEARMLLDNIYLQDGLIASLYRPEADKVAAIEGEFKLRTEQRKTFVELMKRGD  540
AWH65849 - NS3      DEDHAHWLEARMLLDNIYLQDGLIASLYRPEADKVAAIEGEFKLRTEQRKTFVELMKRGD  540
AYI50273 - NS3      DEDHAHWLEARMLLDNIYLQDGLIASLYRPEADKVAAIEGEFKLRTEQRKTFVELMKRGD  540
AYI50274 - NS3      DEDHAHWLEARMLLDNIYLQDGLIASLYRPEADKVAAIEGEFKLRTEQRKTFVELMKRGD  540
AYI50275 - NS3      DEDHAHWLEARMLLDNIYLQDGLIASLYRPEADKVAAIEGEFKLRTEQRKTFVELMKRGD  540
AYI50388 - NS3      DEDHAHWLEARMLLDNIYLQDGLIASLYRPEADKVAAIEGEFKLRTEQRKTFVELMKRGD  540
QCG74166 - NS3      DEDHAHWLEARMLLDNIYLQDGLIASLYRPEADKVAAIEGEFKLRTEQRKTFVELMKRGD  540
YP_002790881 - NS3  DEGHAHWLEARMLLDNIYLQDGLIASLYRPEADKVAAIEGEFKLRTEQRKTFVELMKRGD  540
AEN75265 - NS3      DEDHAHWLEARMLLDNIYLQDGLIASLYRPEADKVAAIEGEFKLRTEQRKTFVELMKRGD  540
AMR68906 - NS3      DEDHAHWLEARMLLDNIYLQDGLIASLYRPEADKVAAIEGEFKLRTEQRKTFVELMKRGD  540

Consensus           LPVWLAYQVASAGITYTDRRWCFDGTTNNTIMEDSVPAEVWTRHGEKRVLKPRWMDARVC  600
AMC13911 - NS3      LPVWLAYQVASAGITYTDRRWCFDGTTNNTIMEDSVPAEVWTRHGEKRVLKPRWMDARVC  600
AMZ03556 - NS3      LPVWLAYQVASAGITYTDRRWCFDGTTNNTIMEDSVPAEVWTRHGEKRVLKPRWMDARVC  600
ANW07476 - NS3      LPVWLAYQVASAGITYTDRRWCFDGTTNNTIMEDSVPAEVWTRHGEKRVLKPRWMDARVC  600
AWH65849 - NS3      LPVWLAYQVASAGITYTDRRWCFDGTTNNTIMEDSVPAEVWTRHGEKRVLKPRWMDARVC  600
AYI50273 - NS3      LPVWLAYQVASAGITYTDRRWCFDGTTNNTIMEDSVPAEVWTRHGEKRVLKPRWMDARVC  600
AYI50274 - NS3      LPVWLAYQVASAGITYTDRRWCFDGTTNNTIMEDSVPAEVWTRHGEKRVLKPRWMDARVC  600
AYI50275 - NS3      LPVWLAYQVASAGITYTDRRWCFDGTTNNTIMEDSVPAEVWTRHGEKRVLKPRWMDARVC  600
AYI50388 - NS3      LPVWLAYQVASAGITYTDRRWCFDGTTNNTIMEDSVPAEVWTRHGEKRVLKPRWMDARVC  600
QCG74166 - NS3      LPVWLAYQVASAGITYTDRRWCFDGTTNNTIMEDSVPAEVWTRHGEKRVLKPRWMDARVC  600
YP_002790881 - NS3  LPVWLAYQVASAGITYTDRRWCFDGTTNNTIMEDSVPAEVWTKYGEKRVLKPRWMDARVC  600
AEN75265 - NS3      LPVWLAYQVASAGITYTDRRWCFDGTTNNTIMEDSVPAEVWTKYGEKRVLKPRWMDARVC  600
AMR68906 - NS3      LPVWLAYQVASAGITYTDRRWCFDGTTNNTIMEDSVPAEVWTKYGEKRVLKPRWMDARVC  600

Consensus           SDHAALKSFKEFAAGKR                                             617
AMC13911 - NS3      SDHAALKSFKEFAAGKR                                             617
AMZ03556 - NS3      SDHAALKSFKEFAAGKR                                             617
ANW07476 - NS3      SDHAALKSFKEFAAGKR                                             617
AWH65849 - NS3      SDHAALKSFKEFAAGKR                                             617
AYI50273 - NS3      SDHAALKSFKEFAAGKR                                             617
AYI50274 - NS3      SDHAALKSFKEFAAGKR                                             617
AYI50275 - NS3      SDHAALKSFKEFAAGKR                                             617
AYI50388 - NS3      SDHAALKSFKEFAAGKR                                             617
QCG74166 - NS3      SDHAALKSFKEFAAGKR                                             617
YP_002790881 - NS3  SDHAALKSFKEFAAGKR                                             617
AEN75265 - NS3      SDHAALKSFKEFAAGKR                                             617
AMR68906 - NS3      SDHAALKSFKEFAAGKR                                             617
```

NS4A

```
Consensus            GAAFGVMEALGTLPGHMTERFQEAIDNLAVLMRAETGSRPYKAAAAQLPETLETIMLLGL   60
AYI50388 - NS4A      GAAFGVMEALGTLPGHMTERFQEAIDNLAVLMRAETGSRPYKAAAAQLPETLETIMLLGL   60
AYI50275 - NS4A      GAAFGVMEALGTLPGHMTERFQEAIDNLAVLMRAETGSRPYKAAAAQLPETLETIMLLGL   60
AWH65849 - NS4A      GAAFGVMEALGTLPGHMTERFQEAIDNLAVLMRAETGSRPYKAAAAQLPETLETIMLLGL   60
AMC13911 - NS4A      GAAFGVMEALGTLPGHMTERFQEAIDNLAVLMRAETGSRPYKAAAAQLPETLETIMLLGL   60
AYI50274 - NS4A      GAAFGVMEALGTLPGHMTERFQEAIDNLAVLMRAETGSRPYKAAAAQLPETLETIMLLGL   60
QCG74166 - NS4A      GAAFGVMEALGTLPGHMTERFQEAIDNLAVLMRAETGSRPYKAAAAQLPETLETIMLLGL   60
ANW07476 - NS4A      GAAFGVMEALGTLPGHMTERFQEAIDNLAVLMRAETGSRPYKAAAAQLPETLETIMLLGL   60
AYI50273 - NS4A      GAAFGVMEALGTLPGHMTERFQEAIDNLAVLMRAETGSRPYKAAAAQLPETLETIMLLGL   60
```

```
AMZ03556 - NS4A     GAAFGVMEALGTLPGHMTERFQEAIDNLAVLMRAETGSRPYKAAAAQLPETLETIMLLGL   60
YP_002790881 - NS4A GAALGVMEALGTLPGHMTERFQEAIDNLAVLMRAETGSRPYKAAAAQLPETLETIMLLGL   60
AEN75265 - NS4A     GAALGVMDALGTLPGHMTERFQEAIDNLAVLMRAETGSRPYKAAAAQLPETLETIMLLGL   60
AMR68906 - NS4A     GAALGVMDALGTLPGHMTERFQEAIDNLAVLMRAETGSRPYKAAAAQLPETLETIMLLGL   60

Consensus           LGTVSLGIFFVLMRNKGIGKMGFGMVTLGASAWLMWLSEIEPARIACVLIVVFLLLVVLI  120
AYI50388 - NS4A     LGTVSLGIFFVLMRNKGIGKMGFGMVTLGASAWLMWLSEIEPARIACVLIVVFLLLVVLI  120
AYI50275 - NS4A     LGTVSLGIFFVLMRNKGIGKMGFGMVTLGASAWLMWLSEIEPARIACVLIVVFLLLVVLI  120
AWH65849 - NS4A     LGTVSLGIFFVLMRNKGIGKMGFGMVTLGASAWLMWLSEIEPARIACVLIVVFLLLVVLI  120
AMC13911 - NS4A     LGTVSLGIFFVLMRNKGIGKMGFGMVTLGASAWLMWLSEIEPARIACVLIVVFLLLVVLI  120
AYI50274 - NS4A     LGTVSLGIFFVLMRNKGIGKMGFGMVTLGASAWLMWLSEIEPARIACVLIVVFLLLVVLI  120
QCG74166 - NS4A     LGTVSLGIFFVLMRNKGIGKMGFGMVTLGASAWLMWLSEIEPARIACVLIVVFLLLVVLI  120
ANW07476 - NS4A     LGTVSLGIFFVLMRNKGIGKMGFGMVTLGASAWLMWLSEIEPARIACVLIVVFLLLVVLI  120
AYI50273 - NS4A     LGTVSLGIFFVLMRNKGIGKMGFGMVTLGASAWLMWLSEIEPARIACVLIVVFLLLVVLI  120
AMZ03556 - NS4A     LGTVSLGIFFVLMRNKGIGKMGFGMVTLGASAWLMWLSEIEPARIACVLIVVFLLLVVLI  120
YP_002790881 - NS4A LGTVSLGIFFVLMRNKGIGKMGFGMVTLGASAWLMWLSEIEPARIACVLIVVFLLLVVLI  120
AEN75265 - NS4A     LGTVSLGIFFVLMRNKGIGKMGFGMVTLGASAWLMWLSEIEPARIACVLIVVFLLLVVLI  120
AMR68906 - NS4A     LGTVSLGIFFVLMRNKGIGKMGFGMVTLGASAWLMWLSEIEPARIACVLIVVFLLLVVLI  120

Consensus           PEPEKQR                                                       127
AYI50388 - NS4A     PEPEKQR                                                       127
AYI50275 - NS4A     PEPEKQR                                                       127
AWH65849 - NS4A     PEPEKQR                                                       127
AMC13911 - NS4A     PEPEKQR                                                       127
AYI50274 - NS4A     PEPEKQR                                                       127
QCG74166 - NS4A     PEPEKQR                                                       127
ANW07476 - NS4A     PEPEKQR                                                       127
AYI50273 - NS4A     PEPEKQR                                                       127
AMZ03556 - NS4A     PEPEKQR                                                       127
YP_002790881 - NS4A PEPEKQR                                                       127
AEN75265 - NS4A     PEPEKQR                                                       127
AMR68906 - NS4A     PEPEKQR                                                       127
```

NS5

```
Consensus          GGGTGETLGEKWKARLNQMSALEFYSYKKSGITEVCREEARRALKDGVATGGHAVSRGSA   60
AMC13911 - NS5     GGGTGETLGEKWKARLNQMSALEFYSYKKSGITEVCREEARRALKDGVATGGHAVSRGSA   60
AMZ03556 - NS5     GGGTGETLGEKWKARLNQMSALEFYSYKKSGITEVCREEARRALKDGVATGGHAVSRGSA   60
ANW07476 - NS5     GGGTGETLGEKWKARLNQMSALEFYSYKKSGITEVCREEARRALKDGVATGGHAVSRGSA   60
AWH65849 - NS5     GGGTGETLGEKWKARLNQMSALEFYSYKKSGITEVCREEARRALKDGVATGGHAVSRGSA   60
AYI50273 - NS5     GGGTGETLGEKWKARLNQMSALEFYSYKKSGITEVCREEARRALKDGVATGGHAVSRGSA   60
AYI50274 - NS5     GGGTGETLGEKWKARLNQMSALEFYSYKKSGITEVCREEARRALKDGVATGGHAVSRGSA   60
AYI50388 - NS5     GGGTGETLGEKWKARLNQMSALEFYSYKKSGITEVCREEARRALKDGVATGGHAVSRGSA   60
QCG74166 - NS5     GGGTGETLGEKWKARLNQMSALEFYSYKKSGITEVCREEARRALKDGVATGGHAVSRGSA   60
AYI50275 - NS5     GGGTGETLGEKWKARLNQMSALEFYSYKKSGITEVCREEARRALKDGVATGGHAVSRGSA   60
YP_002790881 - NS5 GGGTGETLGEKWKARLNQMSALEFYSYKKSGITEVCREEARRALKDGVATGGHAVSRGSA   60
AEN75265 - NS5     GGGTGETLGEKWKARLNQMSALEFYSYKKSGITEVCREEARRALKDGVATGGHAVSRGSA   60
AMR68906 - NS5     GGGTGETLGEKWKARLNQMSALEFYSYKKSGITEVCREEARRALKDGVATGGHAVSRGSA   60

Consensus          KLRWLVERGYLQPYGKVIDLGCGRGGWSYYVATIRKVQEVKGYTKGGPGHEEPVLVQSYG  120
AMC13911 - NS5     KLRWLVERGYLQPYGKVIDLGCGRGGWSYYVATIRKVQEVKGYTKGGPGHEEPVLVQSYG  120
AMZ03556 - NS5     KLRWLVERGYLQPYGKVIDLGCGRGGWSYYVATIRKVQEVKGYTKGGPGHEEPVLVQSYG  120
ANW07476 - NS5     KLRWLVERGYLQPYGKVIDLGCGRGGWSYYVATIRKVQEVKGYTKGGPGHEEPVLVQSYG  120
AWH65849 - NS5     KLRWLVERGYLQPYGKVIDLGCGRGGWSYYVATIRKVQEVKGYTKGGPGHEEPVLVQSYG  120
AYI50273 - NS5     KLRWLVERGYLQPYGKVIDLGCGRGGWSYYVATIRKVQEVKGYTKGGPGHEEPVLVQSYG  120
AYI50274 - NS5     KLRWLVERGYLQPYGKVIDLGCGRGGWSYYVATIRKVQEVKGYTKGGPGHEEPVLVQSYG  120
AYI50388 - NS5     KLRWLVERGYLQPYGKVIDLGCGRGGWSYYVATIRKVQEVKGYTKGGPGHEEPVLVQSYG  120
QCG74166 - NS5     KLRWLVERGYLQPYGKVIDLGCGRGGWSYYVATIRKVQEVKGYTKGGPGHEEPVLVQSYG  120
AYI50275 - NS5     KLRWLVERGYLQPYGKVIDLGCGRGGWSYYVATIRKVQEVKGYTKGGPGHEEPVLVQSYG  120
YP_002790881 - NS5 KIRWLEERGYLQPYGKVVDLGCGRGGWSYYAATIRKVQEVRGYTKGGPGHEEPMLVQSYG  120
AEN75265 - NS5     KLRWLVERGYLQPHGKVVDLGCGRGGWSYYAATIRKVQEVRGYTKGGPGHEEPMLVQSYG  120
AMR68906 - NS5     KLRWLVERGYLQPHGKVVDLGCGRGGWSYYAATIRKVQEVRGYTKGGPGHEEPMLVQSYG  120
```

```
Consensus           WNIVRLKSGVDVFHMAAEPCDTLLCDIGESSSSPEVEEARTLRVLSMVGDWLEKRPGAFC  180
AMC13911 - NS5      WNIVRLKSGVDVFHMAAEPCDTLLCDIGESSSSPEVEEARTLRVLSMVGDWLEKRPGAFC  180
AMZ03556 - NS5      WNIVRLKSGVDVFHMAAEPCDTLLCDIGESSSSPEVEEARTLRVLSMVGDWLEKRPGAFC  180
ANW07476 - NS5      WNIVRLKSGVDVFHMAAEPCDTLLCDIGESSSSPEVEEARTLRVLSMVGDWLEKRPGAFC  180
AWH65849 - NS5      WNIVRLKSGVDVFHMAAEPCDTLLCDIGESSSSPEVEEARTLRVLSMVGDWLEKRPGAFC  180
AYI50273 - NS5      WNIVRLKSGVDVFHMAAEPCDTLLCDIGESSSSPEVEEARTLRVLSMVGDWLEKRPGAFC  180
AYI50274 - NS5      WNIVRLKSGVDVFHMAAEPCDTLLCDIGESSSSPEVEEARTLRVLSMVGDWLEKRPGAFC  180
AYI50388 - NS5      WNIVRLKSGVDVFHMAAEPCDTLLCDIGESSSSPEVEEARTLRVLSMVGDWLEKRPGAFC  180
QCG74166 - NS5      WNIVRLKSGVDVFHMAAEPCDTLLCDIGESSSSPEVEEARTLRVLSMVGDWLEKRPGAFC  180
AYI50275 - NS5      WNIVRLKSGVDVFHMAAEPCDTLLCDIGESSSSPEVEEARTLRVLSMVGDWLEKRPGAFC  180
YP_002790881 - NS5  WNIVRLKSGVDVFHMAAEPCDTLLCDIGESSSSPEVEETRTLRVLSMVGDWLEKRPGAFC  180
AEN75265 - NS5      WNIVRLKSGVDVFHMAAEPCDTLLCDIGESSSSPEVEETRTLRVLSMVGDWLEKRPGAFC  180
AMR68906 - NS5      WNIVRLKSGVDVFHMAAEPCDTLLCDIGESSSSPEVEETRTLRVLSMVGDWLEKRPGAFC  180

Consensus           IKVLCPYTSTMMETLERLQRRYGGGLVRVPLSRNSTHEMYWVSGAKSNTIKSVSTTSQLL  240
AMC13911 - NS5      IKVLCPYTSTMMETLERLQRRYGGGLVRVPLSRNSTHEMYWVSGAKSNTIKSVSTTSQLL  240
AMZ03556 - NS5      IKVLCPYTSTMMETLERLQRRYGGGLVRVPLSRNSTHEMYWVSGAKSNTIKSVSTTSQLL  240
ANW07476 - NS5      IKVLCPYTSTMMETLERLQRRYGGGLVRVPLSRNSTHEMYWVSGAKSNTIKSVSTTSQLL  240
AWH65849 - NS5      IKVLCPYTSTMMETLERLQRRYGGGLVRVPLSRNSTHEMYWVSGAKSNTIKSVSTTSQLL  240
AYI50273 - NS5      IKVLCPYTSTMMETLERLQRRYGGGLVRVPLSRNSTHEMYWVSGAKSNTIKSVSTTSQLL  240
AYI50274 - NS5      IKVLCPYTSTMMETLERLQRRYGGGLVRVPLSRNSTHEMYWVSGAKSNTIKSVSTTSQLL  240
AYI50388 - NS5      IKVLCPYTSTMMETLERLQRRYGGGLVRVPLSRNSTHEMYWVSGAKSNTIKSVSTTSQLL  240
QCG74166 - NS5      IKVLCPYTSTMMETLERLQRRYGGGLVRVPLSRNSTHEMYWVSGAKSNTIKSVSTTSQLL  240
AYI50275 - NS5      IKVLCPYTSTMMETLERLQRRYGGGLVRVPLSRNSTHEMYWVSGAKSNTIKSVSTTSQLL  240
YP_002790881 - NS5  IKVLCPYTSTMMETMERLQRRHGGGLVRVPLCRNSTHEMYWVSGAKSNIIKSVSTTSQLL  240
AEN75265 - NS5      IKVLCPYTSTMMETMERLQRRYGGGLVRVPLSRNSTHEMYWVSGAKSNIIKSVSTTSQLL  240
AMR68906 - NS5      IKVLCPYTSTMMETMERLQRRYGGGLVRVPLSRNSTHEMYWVSGAKSNIIKSVSTTSQLL  240

Consensus           LGRMDGPRRPVKYEEDVNLGSGTRAVVSCAEAPNMKIIGNRIERIRSEHAETWFFDENHP  300
AMC13911 - NS5      LGRMDGPRRPVKYEEDVNLGSGTRAVVSCAEAPNMKIIGNRIERIRSEHAETWFFDENHP  300
AMZ03556 - NS5      LGRMDGPRRPVKYEEDVNLGSGTRAVVSCAEAPNMKIIGNRIERIRSEHAETWFFDENHP  300
ANW07476 - NS5      LGRMDGPRRPVKYEEDVNLGSGTRAVVSCAEAPNMKIIGNRIERIRSEHAETWFFDENHP  300
AWH65849 - NS5      LGRMDGPRRPVKYEEDVNLGSGTRAVVSCAEAPNMKIIGNRIERIRSEHAETWFFDENHP  300
AYI50273 - NS5      LGRMDGPRRPVKYEEDVNLGSGTRAVVSCAEAPNMKIIGNRIERIRSEHAETWFFDENHP  300
AYI50274 - NS5      LGRMDGPRRPVKYEEDVNLGSGTRAVVSCAEAPNMKIIGNRIERIRSEHAETWFFDENHP  300
AYI50388 - NS5      LGRMDGPRRPVKYEEDVNLGSGTRAVVSCAEAPNMKIIGNRIERIRSEHAETWFFDENHP  300
QCG74166 - NS5      LGRMDGPRRPVKYEEDVNLGSGTRAVVSCAEAPNMKIIGNRIERIRSEHAETWFFDENHP  300
AYI50275 - NS5      LGRMDGPRRPVKYEEDVNLGSGTRAVVSCAEAPNMKIIGNRIERIRSEHAETWFFDENHP  300
YP_002790881 - NS5  LGRMDGPRRPVKYEEDVNLGSGTRAVASCAEAPNMKIIGRRIERIRNEHAETWFLDENHP  300
AEN75265 - NS5      LGRMDGPRRPVKYEEDVNLGSGTRAVASCAEAPNMKIIGRRIERIRNEHAETWFFDENHP  300
AMR68906 - NS5      LGRMDGPRRPVKYEEDVNLGSGTRAVASCAEAPNMKIIGRRIERIRNEHAETWFFDENHP  300

Consensus           YRTWAYHGSYEAPTQGSASSLINGVVRLLSKPWDVVTGVTGIAMTDTTPYGQQRVFKEKV  360
AMC13911 - NS5      YRTWAYHGSYEAPTQGSASSLINGVVRLLSKPWDVVTGVTGIAMTDTTPYGQQRVFKEKV  360
AMZ03556 - NS5      YRTWAYHGSYEAPTQGSASSLINGVVRLLSKPWDVVTGVTGIAMTDTTPYGQQRVFKEKV  360
ANW07476 - NS5      YRTWAYHGSYEAPTQGSASSLINGVVRLLSKPWDVVTGVTGIAMTDTTPYGQQRVFKEKV  360
AWH65849 - NS5      YRTWAYHGSYEAPTQGSASSLINGVVRLLSKPWDVVTGVTGIAMTDTTPYGQQRVFKEKV  360
AYI50273 - NS5      YRTWAYHGSYEAPTQGSASSLINGVVRLLSKPWDVVTGVTGIAMTDTTPYGQQRVFKEKV  360
AYI50274 - NS5      YRTWAYHGSYEAPTQGSASSLINGVVRLLSKPWDVVTGVTGIAMTDTTPYGQQRVFKEKV  360
AYI50388 - NS5      YRTWAYHGSYEAPTQGSASSLINGVVRLLSKPWDVVTGVTGIAMTDTTPYGQQRVFKEKV  360
QCG74166 - NS5      YRTWAYHGSYEAPTQGSASSLINGVVRLLSKPWDVVTGVTGIAMTDTTPYGQQRVFKEKV  360
AYI50275 - NS5      YRTWAYHGSYEAPTQGSASSLINGVVRLLSKPWDVVTGVTGIAMTDTTPYGQQRVFKEKV  360
YP_002790881 - NS5  YRTWAYHGSYEAPTQGSASSLVNGVVRLLSKPWDVVTGVTGIAMTDTTPYGQQRVFKEKV  360
AEN75265 - NS5      YRTWAYHGSYEAPTQGSASSLVNGVVRLLSKPWDVVTGVTGIAMTDTTPYGQQRVFKEKV  360
AMR68906 - NS5      YRTWAYHGSYEAPTQGSASSLVNGVVRLLSKPWDVVTGVTGIAMTDTTPYGQQRVFKEKV  360

Consensus           DTRVPDPQEGTRQVMSMVSSWLWKELGKHKRPRVCTKEEFINKVRSNAALGAIFEEEKEW  420
AMC13911 - NS5      DTRVPDPQEGTRQVMSMVSSWLWKELGKHKRPRVCTKEEFINKVRSNAALGAIFEEEKEW  420
AMZ03556 - NS5      DTRVPDPQEGTRQVMSMVSSWLWKELGKHKRPRVCTKEEFINKVRSNAALGAIFEEEKEW  420
ANW07476 - NS5      DTRVPDPQEGTRQVMSMVSSWLWKELGKHKRPRVCTKEEFINKVRSNAALGAIFEEEKEW  420
AWH65849 - NS5      DTRVPDPQEGTRQVMSMVSSWLWKELGKHKRPRVCTKEEFINKVRSNAALGAIFEEEKEW  420
AYI50273 - NS5      DTRVPDPQEGTRQVMSMVSSWLWKELGKHKRPRVCTKEEFINKVRSNAALGAIFEEEKEW  420
AYI50274 - NS5      DTRVPDPQEGTRQVMSMVSSWLWKELGKHKRPRVCTKEEFINKVRSNAALGAIFEEEKEW  420
```

```
AYI50388 - NS5      DTRVPDPQEGTRQVMSMVSSWLWKELGKHKRPRVCTKEEFINKVRSNAALGAIFEEEKEW  420
QCG74166 - NS5      DTRVPDPQEGTRQVMSMVSSWLWKELGKHKRPRVCTKEEFINKVRSNAALGAIFEEEKEW  420
AYI50275 - NS5      DTRVPDPQEGTRQVMSMVSSWLWKELGKHKRPRVCTKEEFINKVRSNAALGAIFEEEKEW  420
YP_002790881 - NS5  DTRVPDPQEGTRQVMNIVSSWLWKELGKRKRPRVCTKEEFINKVRSNAALGAIFEEEKEW  420
AEN75265 - NS5      DTRVPDPQEGTRRVMNMVSSWLWKELGKRKRPRVCTKEEFINKVRSNAALGAIFEEEKEW  420
AMR68906 - NS5      DTRVPDPQEGTRRVMNMVSSWLWKELGKRKRPRVCTKEEFINKVRSNAALGAIFEEEKEW  420

Consensus           KTAVEAVNDPRFWALVDKEREHHLRGECQSCVYNMMGKREKKQGEFGKAKGSRAIWYMWL  480
AMC13911 - NS5      KTAVEAVNDPRFWALVDKEREHHLRGECQSCVYNMMGKREKKQGEFGKAKGSRAIWYMWL  480
AMZ03556 - NS5      KTAVEAVNDPRFWALVDKEREHHLRGECQSCVYNMMGKREKKQGEFGKAKGSRAIWYMWL  480
ANW07476 - NS5      KTAVEAVNDPRFWALVDKEREHHLRGECQSCVYNMMGKREKKQGEFGKAKGSRAIWYMWL  480
AWH65849 - NS5      KTAVEAVNDPRFWALVDKEREHHLRGECQSCVYNMMGKREKKQGEFGKAKGSRAIWYMWL  480
AYI50273 - NS5      KTAVEAVNDPRFWALVDKEREHHLRGECQSCVYNMMGKREKKQGEFGKAKGSRAIWYMWL  480
AYI50274 - NS5      KTAVEAVNDPRFWALVDKEREHHLRGECQSCVYNMMGKREKKQGEFGKAKGSRAIWYMWL  480
AYI50388 - NS5      KTAVEAVNDPRFWALVDKEREHHLRGECQSCVYNMMGKREKKQGEFGKAKGSRAIWYMWL  480
QCG74166 - NS5      KTAVEAVNDPRFWALVDKEREHHLRGECQSCVYNMMGKREKKQGEFGKAKGSRAIWYMWL  480
AYI50275 - NS5      KTAVEAVNDPRFWALVDKEREHHLRGECQSCVYNMMGKREKKQGEFGKAKGSRAIWYMWL  480
YP_002790881 - NS5  KTAVEAVNDPRFWALVDREREHHLRGECHSCVYNMMGKREKKQGEFGKAKGSRAIWYMWL  480
AEN75265 - NS5      KTAVEAVNDPRFWALVDKEREHHLRGECHSCVYNMMGKREKKQGEFGKAKGSRAIWYMWL  480
AMR68906 - NS5      KTAVEAVNDPRFWALVDKEREHHLRGECHSCVYNMMGKREKKQGEFGKAKGSRAIWYMWL  480

Consensus           GARFLEFEALGFLNEDHWMGRENSGGGVEGLGLQRLGYVLEEMSRIPGGRMYADDTAGWD  540
AMC13911 - NS5      GARFLEFEALGFLNEDHWMGRENSGGGVEGLGLQRLGYVLEEMSRIPGGRMYADDTAGWD  540
AMZ03556 - NS5      GARFLEFEALGFLNEDHWMGRENSGGGVEGLGLQRLGYVLEEMSRIPGGRMYADDTAGWD  540
ANW07476 - NS5      GARFLEFEALGFLNEDHWMGRENSGGGVEGLGLQRLGYVLEEMSRIPGGRMYADDTAGWD  540
AWH65849 - NS5      GARFLEFEALGFLNEDHWMGRENSGGGVEGLGLQRLGYVLEEMSRIPGGRMYADDTAGWD  540
AYI50273 - NS5      GARFLEFEALGFLNEDHWMGRENSGGGVEGLGLQRLGYVLEEMSRIPGGRMYADDTAGWD  540
AYI50274 - NS5      GARFLEFEALGFLNEDHWMGRENSGGGVEGLGLQRLGYVLEEMSRIPGGRMYADDTAGWD  540
AYI50388 - NS5      GARFLEFEALGFLNEDHWMGRENSGGGVEGLGLQRLGYVLEEMSRIPGGRMYADDTAGWD  540
QCG74166 - NS5      GARFLEFEALGFLNEDHWMGRENSGGGVEGLGLQRLGYVLEEMSRIPGGRMYADDTAGWD  540
AYI50275 - NS5      GARFLEFEALGFLNEDHWMGRENSGGGVEGLGLQRLGYVLEEMSRIPGGRMYADDTAGWD  540
YP_002790881 - NS5  GARFLEFEALGFLNEDHWMGRENSGGGVEGLGLQRLGYILEEMNRAPGGKMYADDTAGWD  540
AEN75265 - NS5      GARFLEFEALGFLNEDHWMGRENSGGGVEGLGLQRLGYILEEMNRAPGGKMYADDTAGWD  540
AMR68906 - NS5      GARFLEFEALGFLNEDHWMGRENSGGGVEGLGLQRLGYILEEMNRAPGGKMYADDTAGWD  540

Consensus           TRISRFDLENEALITNQMEKGHRALALAIIKYTYQNKVVKVLRPAEKGKTVMDIISRQDQ  600
AMC13911 - NS5      TRISRFDLENEALITNQMEKGHRALALAIIKYTYQNKVVKVLRPAEKGKTVMDIISRQDQ  600
AMZ03556 - NS5      TRISRFDLENEALITNQMEKGHRALALAIIKYTYQNKVVKVLRPAEKGKTVMDIISRQDQ  600
ANW07476 - NS5      TRISRFDLENEALITNQMEKGHRALALAIIKYTYQNKVVKVLRPAEKGKTVMDIISRQDQ  600
AWH65849 - NS5      TRISRFDLENEALITNQMEKGHRALALAIIKYTYQNKVVKVLRPAEKGKTVMDIISRQDQ  600
AYI50273 - NS5      TRISRFDLENEALITNQMEKGHRALALAIIKYTYQNKVVKVLRPAEKGKTVMDIISRQDQ  600
AYI50274 - NS5      TRISRFDLENEALITNQMEKGHRALALAIIKYTYQNKVVKVLRPAEKGKTVMDIISRQDQ  600
AYI50388 - NS5      TRISRFDLENEALITNQMEKGHRALALAIIKYTYQNKVVKVLRPAEKGKTVMDIISRQDQ  600
QCG74166 - NS5      TRISRFDLENEALITNQMEKGHRALALAIIKYTYQNKVVKVLRPAEKGKTVMDIISRQDQ  600
AYI50275 - NS5      TRISRFDLENEALITNQMEKGHRALALAIIKYTYQNKVVKVLRPAEKGKTVMDIISRQDQ  600
YP_002790881 - NS5  TRISKFDLENEALITNQMEEGHRTLALAVIKYTYQNKVVKVLRPAEGGKTVMDIISRQDQ  600
AEN75265 - NS5      TRISRFDLENEALITNQMEEGHRALALAVIKYTYQNKVVKVLRPAEGGKTVMDIISRQDQ  600
AMR68906 - NS5      TRISRFDLENEALITNQMEEGHRALALAVIKYTYQNKVVKVLRPAEGGKTVMDIISRQDQ  600

Consensus           RGSGQVVTYALNTFTNLVVQLIRNMEAEEVLEMQDLWLLRRSEKVTNWLQSNGWDRLKRM  660
AMC13911 - NS5      RGSGQVVTYALNTFTNLVVQLIRNMEAEEVLEMQDLWLLRRSEKVTNWLQSNGWDRLKRM  660
AMZ03556 - NS5      RGSGQVVTYALNTFTNLVVQLIRNMEAEEVLEMQDLWLLRRSEKVTNWLQSNGWDRLKRM  660
ANW07476 - NS5      RGSGQVVTYALNTFTNLVVQLIRNMEAEEVLEMQDLWLLRRSEKVTNWLQSNGWDRLKRM  660
AWH65849 - NS5      RGSGQVVTYALNTFTNLVVQLIRNMEAEEVLEMQDLWLLRRSEKVTNWLQSNGWDRLKRM  660
AYI50273 - NS5      RGSGQVVTYALNTFTNLVVQLIRNMEAEEVLEMQDLWLLRRSEKVTNWLQSNGWDRLKRM  660
AYI50274 - NS5      RGSGQVVTYALNTFTNLVVQLIRNMEAEEVLEMQDLWLLRRSEKVTNWLQSNGWDRLKRM  660
AYI50388 - NS5      RGSGQVVTYALNTFTNLVVQLIRNMEAEEVLEMQDLWLLRRSEKVTNWLQSNGWDRLKRM  660
QCG74166 - NS5      RGSGQVVTYALNTFTNLVVQLIRNMEAEEVLEMQDLWLLRRSEKVTNWLQSNGWDRLKRM  660
AYI50275 - NS5      RGSGQVVTYALNTFTNLVVQLIRNMEAEEVLEMQDLWLLRRSEKVTNWLQSNGWDRLKRM  660
YP_002790881 - NS5  RGSGQVVTYALNTFTNLVVQLIRNMEAEEVLEMQDLWLLRKPEKVTRWLQSNGWDRLKRM  660
AEN75265 - NS5      RGSGQVVTYALNTFTNLVVQLIRNMEAEEVLEMHDLWLLRKPEKVTRWLQSNGWDRLKRM  660
AMR68906 - NS5      RGSGQVVTYALNTFTNLVVQLIRNMEAEEVLEMHDLWLLRKPEKVTRWLQSNGWDRLKRM  660
```

```
Consensus          AVSGDDCVVKPIDDRFAHALRFLNDMGKVRKDTQEWKPSTGWDNWEEVPFCSHHFNKLHL  720
AMC13911 - NS5     AVSGDDCVVKPIDDRFAHALRFLNDMGKVRKDTQEWKPSTGWDNWEEVPFCSHHFNKLHL  720
AMZ03556 - NS5     AVSGDDCVVKPIDDRFAHALRFLNDMGKVRKDTQEWKPSTGWDNWEEVPFCSHHFNKLHL  720
ANW07476 - NS5     AVSGDDCVVKPIDDRFAHALRFLNDMGKVRKDTQEWKPSTGWDNWEEVPFCSHHFNKLHL  720
AWH65849 - NS5     AVSGDDCVVKPIDDRFAHALRFLNDMGKVRKDTQEWKPSTGWDNWEEVPFCSHHFNKLHL  720
AYI50273 - NS5     AVSGDDCVVKPIDDRFAHALRFLNDMGKVRKDTQEWKPSTGWDNWEEVPFCSHHFNKLHL  720
AYI50274 - NS5     AVSGDDCVVKPIDDRFAHALRFLNDMGKVRKDTQEWKPSTGWDNWEEVPFCSHHFNKLHL  720
AYI50388 - NS5     AVSGDDCVVKPIDDRFAHALRFLNDMGKVRKDTQEWKPSTGWDNWEEVPFCSHHFNKLHL  720
QCG74166 - NS5     AVSGDDCVVKPIDDRFAHALRFLNDMGKVRKDTQEWKPSTGWDNWEEVPFCSHHFNKLHL  720
AYI50275 - NS5     AVSGDDCVVKPIDDRFAHALRFLNDMGKVRKDTQEWKPSTGWDNWEEVPFCSHHFNKLHL  720
YP_002790881 - NS5 AVSGDDCVVKPIDDRFAHALRFLNDMGKVRKDTQEWKPSTGWSNWEEVPFCSHHFNKLYL  720
AEN75265 - NS5     AVSGDDCVVKPIDDRFAHALRFLNDMGKVRKDTQEWKPSTGWSNWEEVPFCSHHFNKLHL  720
AMR68906 - NS5     AVSGDDCVVKPIDDRFAHALRFLNDMGKVRKDTQEWKPSTGWSNWEEVPFCSHHFNKLHL  720

Consensus          KDGRSIVVPCRHQDELIGRARVSPGAGWSIRETACLAKSYAQMWQLLYFHRRDLRLMANA  780
AMC13911 - NS5     KDGRSIVVPCRHQDELIGRARVSPGAGWSIRETACLAKSYAQMWQLLYFHRRDLRLMANA  780
AMZ03556 - NS5     KDGRSIVVPCRHQDELIGRARVSPGAGWSIRETACLAKSYAQMWQLLYFHRRDLRLMANA  780
ANW07476 - NS5     KDGRSIVVPCRHQDELIGRARVSPGAGWSIRETACLAKSYAQMWQLLYFHRRDLRLMANA  780
AWH65849 - NS5     KDGRSIVVPCRHQDELIGRARVSPGAGWSIRETACLAKSYAQMWQLLYFHRRDLRLMANA  780
AYI50273 - NS5     KDGRSIVVPCRHQDELIGRARVSPGAGWSIRETACLAKSYAQMWQLLYFHRRDLRLMANA  780
AYI50274 - NS5     KDGRSIVVPCRHQDELIGRARVSPGAGWSIRETACLAKSYAQMWQLLYFHRRDLRLMANA  780
AYI50388 - NS5     KDGRSIVVPCRHQDELIGRARVSPGAGWSIRETACLAKSYAQMWQLLYFHRRDLRLMANA  780
QCG74166 - NS5     KDGRSIVVPCRHQDELIGRARVSPGAGWSIRETACLAKSYAQMWQLLYFHRRDLRLMANA  780
AYI50275 - NS5     KDGRSIVVPCRHQDELIGRARVSPGAGWSIRETACLAKSYAQMWQLLYFHRRDLRLMANA  780
YP_002790881 - NS5 KDGRSIVVPCRHQDELIGRARVSPGAGWSIRETACLAKSYAQMWQLLYFHRRDLRLMANA  780
AEN75265 - NS5     KDGRSIVVPCRHQDELIGRARVSPGAGWSIRETACLAKSYAQMWQLLYFHRRDLRLMANA  780
AMR68906 - NS5     KDGRSIVVPCRHQDELIGRARVSPGAGWSIRETACLAKSYAQMWQLLYFHRRDLRLMANA  780

Consensus          ICSSVPVDWVPTGRTTWSIHGKGEWMTTEDMLVVWNRVWIEENDHMEDKTPVTKWTDIPY  840
AMC13911 - NS5     ICSSVPVDWVPTGRTTWSIHGKGEWMTTEDMLVVWNRVWIEENDHMEDKTPVTKWTDIPY  840
AMZ03556 - NS5     ICSSVPVDWVPTGRTTWSIHGKGEWMTTEDMLVVWNRVWIEENDHMEDKTPVTKWTDIPY  840
ANW07476 - NS5     ICSSVPVDWVPTGRTTWSIHGKGEWMTTEDMLVVWNRVWIEENDHMEDKTPVTKWTDIPY  840
AWH65849 - NS5     ICSSVPVDWVPTGRTTWSIHGKGEWMTTEDMLVVWNRVWIEENDHMEDKTPVTKWTDIPY  840
AYI50273 - NS5     ICSSVPVDWVPTGRTTWSIHGKGEWMTTEDMLVVWNRVWIEENDHMEDKTPVTKWTDIPY  840
AYI50274 - NS5     ICSSVPVDWVPTGRTTWSIHGKGEWMTTEDMLVVWNRVWIEENDHMEDKTPVTKWTDIPY  840
AYI50388 - NS5     ICSSVPVDWVPTGRTTWSIHGKGEWMTTEDMLVVWNRVWIEENDHMEDKTPVTKWTDIPY  840
QCG74166 - NS5     ICSSVPVDWVPTGRTTWSIHGKGEWMTTEDMLVVWNRVWIEENDHMEDKTPVTKWTDIPY  840
AYI50275 - NS5     ICSSVPVDWVPTGSTTWSIHGKGEWMTTEDMLVVWNRVWIEENDHMEDKTPVTKWTDIPY  840
YP_002790881 - NS5 ICSAVPVDWVPTGRTTWSIHGKGEWMTTEDMLMVWNRVWIEENDHMEDKTPVTKWTDIPY  840
AEN75265 - NS5     ICSAVPADWVPTGRTTWSIHGKGEWMTNEDMLMVWNRVWIEENDHMGDKTPVTKWTDIPY  840
AMR68906 - NS5     ICSAVPADWVPTGRTTWSIHGKGEWMTNEDMLMVWNRVWIEENDHMGDKTPVTKWTDIPY  840

Consensus          LGKREDLWCGSLIGHRPRTTWAENIKNTVNMVRRIIGDEEKYMDYLSTQVRYLGEEGSTP  900
AMC13911 - NS5     LGKREDLWCGSLIGHRPRTTWAENIKNTVNMVRRIIGDEEKYMDYLSTQVRYLGEEGSTP  900
AMZ03556 - NS5     LGKREDLWCGSLIGHRPRTTWAENIKNTVNMVRRIIGDEEKYMDYLSTQVRYLGEEGSTP  900
ANW07476 - NS5     LGKREDLWCGSLIGHRPRTTWAENIKNTVNMVRRIIGDEEKYMDYLSTQVRYLGEEGSTP  900
AWH65849 - NS5     LGKREDLWCGSLIGHRPRTTWAENIKNTVNMVRRIIGDEEKYMDYLSTQVRYLGEEGSTP  900
AYI50273 - NS5     LGKREDLWCGSLIGHRPRTTWAENIKNTVNMVRRIIGDEEKYMDYLSTQVRYLGEEGSTP  900
AYI50274 - NS5     LGKREDLWCGSLIGHRPRTTWAENIKNTVNMVRRIIGDEEKYMDYLSTQVRYLGEEGSTP  900
AYI50388 - NS5     LGKREDLWCGSLIGHRPRTTWAENIKNTVNMVRRIIGDEEKYMDYLSTQVRYLGEEGSTP  900
QCG74166 - NS5     LGKREDLWCGSLIGHRPRTTWAENIKNTVNMVRRIIGDEEKYMDYLSTQVRYLGEEGSTP  900
AYI50275 - NS5     LGKREDLWCGSLIGHRPRTTWAENIKNTVNMVRRIIGDEEKYMDYLSTQVRYLGEEGSTP  900
YP_002790881 - NS5 LGKREDLWCGSLIGHRPRTTWAENIKDTVNMVRRIIGDEEKYMDYLSTQVRYLGEEGSTP  900
AEN75265 - NS5     LGKREDLWCGSLIGHRPRTTWAENIKDTVNMVRRIIGDEERYMDYLSTQVRYLGEEGSTP  900
AMR68906 - NS5     LGKREDLWCGSLIGHRPRTTWAENIKDTVNMVRRIIGDEERYMDYLSTQVRYLGEEGSTP  900

Consensus          GVL                                                           903
AMC13911 - NS5     GVL                                                           903
AMZ03556 - NS5     GVL                                                           903
ANW07476 - NS5     GVL                                                           903
AWH65849 - NS5     GVL                                                           903
AYI50273 - NS5     GVL                                                           903
AYI50274 - NS5     GVL                                                           903
```

```
AYI50388 - NS5     GVL                                                   903
QCG74166 - NS5     GVL                                                   903
AYI50275 - NS5     GVL                                                   903
YP_002790881 - NS5 GVL                                                   903
AEN75265 - NS5     GVL                                                   903
AMR68906 - NS5     GVL                                                   903
```
